# Integrating transcriptome and metabolome to reveal the characteristics of gene expression profiles and metabolite changes of *sorghum* in response to cadmium treatment

**DOI:** 10.3389/fpls.2025.1678876

**Published:** 2025-09-18

**Authors:** Lixi Deng, Yinhua Chen, Yanlin An, Feng Zhang, Ping Zhou, Chenghong Mou, Yuyu Chen

**Affiliations:** ^1^ School of Food Engineering, Moutai Institute, Renhuai, Guizhou, China; ^2^ Department of Scientific Research, Moutai Institute, Renhuai, Guizhou, China; ^3^ School of Resources and Environment, Moutai Institute, Renhuai, Guizhou, China

**Keywords:** cadmium stress, metabolomics detection, qRT-PCR, *sorghum*, transcriptome sequencing, WGCNA

## Abstract

**Introduction:**

*Sorghum* is one of the most important grain crops and industrial crops, and its planting environment is at risk of heavy metal pollution such as cadmium. However, the gene expression characteristics and changes in metabolic flux of *sorghum* in response to cadmium stress still need to be further revealed.

**Methods:**

The glutinous *sorghum* variety “HongYingZi” was treated with 200 μM of cadmium acetate, and samples were collected at a total of eight time points (including the control group and 3h, 6h, 9h, 12h, 24h, 48h, and 72h after treatment) for transcriptome sequencing. The original sequencing data was filtered, aligned, assembled for transcripts, and identified for differentially expressed genes using software such as fastp, Hisat2, StringTie, and DEGseq. Meanwhile, UPLC-MS/MS was used to detect metabolites in all samples. Co-expression analysis was performed using the WGCNA R package, and the expression levels of related genes were verified by qRT-PCR.

**Results:**

In this study, transcriptome sequencing and metabolome analysis were performed on *sorghum* samples treated with cadmium. The results showed that a total of 2,299 differentially expressed genes were identified among different samples, including important transcription factors such as AP2/ERF, bHLH and MYB-related families. Although the number of differential genes among different groups varied greatly from 171 (CK vs TM3h) to 1,410 (CK vs TM9h), these differential genes were mainly significantly enriched in pathways such as metabolic pathways, arachidonic acid metabolism, and starch and sucrose metabolism. Meanwhile, metabolome detection identified 4,656 metabolites in 21 major categories. Although the total content of metabolites showed no significant difference among different samples, 68 amino acids and their derivatives, 16 flavonoids, 21 alkaloids, 92 organic acids and other differential metabolites were identified. Twelve gene modules related to amino acid content were identified through WGCNA analysis. qRT-PCR analysis showed that the expression levels of these genes were highly consistent with the transcriptome results.

**Discussion:**

Our research reveals the gene expression profiles and metabolite change characteristics of glutinous *sorghum* in response to cadmium treatment, which provided data support and new insights for further revealing the genetic mechanism and metabolic flux basis of *sorghum* in response to cadmium.

## 1 Introduction

*Sorghum bicolor* [L.] Moench (2n = 20) is not only one of the most important food crops widely cultivated worldwide, but also widely used in industries such as wine-making, feed production, and fuel preparation. Therefore, in recent years, the planting area of *sorghum* has been gradually expanding, and in 2024, the global *sorghum* output has approached 61.3 million metric tons (https://www.fas.usda.gov/data/production/commodity/0459200) (Tao et al., 2021; Gladman et al., 2022). Meanwhile, due to its excellent characteristics of tolerance to adverse stresses such as drought and salinity, *sorghum* is also widely used as a model crop in the research of gramineae (Sudhakar Reddy et al., 2016). With the advancement of sequencing technologies, several population resequencing projects and high-quality T2T-level *sorghum* reference genomes have been successively released (Tao et al., 2021; Bao et al., 2024; Wei et al., 2024). This has further deepened people’s understanding of *sorghum* breeding and broadened research perspectives. Currently, the demand for food continues to expand due to population growth, while the increasingly frequent occurrences of extreme droughts, floods, and high temperatures undoubtedly pose a huge threat to global food production. The *sorghum* breeding and production also face threats from soil salinization, heavy metal pollution, insect pests, and fungal diseases.

Cadmium (Cd) is one of the most serious soil pollutants in worldwide including in China. As large amounts of Cd are emitted into the environment, it can be easily absorbed by plants and enter the food chain, thereby affecting food security (Wang et al., 2015). Numerous studies have indicated that Cd can enter the human body through the ingestion of contaminated food and beverages (Rasin et al., 2025). A portion of it will be retained in the liver and kidneys, and its excretion is very slow. This results in a half-life as long as 10 to 30 years and increases the risk of lung cancer, endometrial cancer, bladder cancer, and breast cancer (Butaciu et al., 2016). Therefore, studies on Cd uptake, transport, and resistance to Cd stress have been carried out in many plants (Guo and Chi, 2014; Haider et al., 2021; Shan et al., 2025). For example, researchers not only studied the accumulation and translocation patterns of cadmium in maize seedlings under different Cd concentrations, but also identified a single nucleotide polymorphism (SNP, SYN25051) located on chromosome 2 of maize that is closely associated with Cd content in leaves through a genome-wide association study (GWAS) analysis (Nguyen et al., 2016; Zhao et al., 2018). Rice is generally regarded as a plant with high Cd accumulation. In East Asia and Southeast Asia, the amount of Cd ingested through rice can reach 20-40μg per person per day, which is much higher than the limit of European Food Safety Agency (Cd < 2.5μg/d) (Jing et al., 2023). The results show that there are four main processes of Cd absorption by rice, including root absorption and xylem loading-mediated transport to the aboveground (Li et al., 2017). In addition, the membrane transporter family has also been shown to be involved in the uptake, translocation, detoxification, and distribution of cadmium into various organelles (Tao and Lu, 2022).

In previous studies, many researchers have thoroughly explored the genetic regulatory mechanisms of *sorghum* in response to various stresses, such as drought, salinity-alkalinity, high temperature, and pests and diseases (Zheng et al., 2024). It has been found that members of the HD-ZIP and bZIP transcription factor families are involved in the synergistic mechanism of drought and salt tolerance in *sorghum* (Li et al., 2024b); whereas the *SbMYBHv33* gene can negatively regulate biomass accumulation and salt tolerance in *sorghum* (Zheng et al., 2023). Through comparative analysis of two sweet *sorghum* genotypes with significant differences (high Cd-accumulating genotype H18 and low Cd-accumulating genotype L69), it was found that the higher Cd accumulation in H18 depends on the multi-level coordination of efficient Cd uptake and translocation, including efficient root uptake and xylem loading, less root cell wall binding, and a weaker endodermal apoplastic barrier (Feng et al., 2018). Analysis of the Cd tolerance, uptake and accumulation capacities of 96 *sorghum* varieties revealed that significant differences may exist among different *sorghum* varieties in these three aspects (Jia et al., 2017). However, studies on Cd in *sorghum* have mainly used sweet *sorghum* as the material, while the transcriptional regulatory mechanisms and changes in metabolic flux of glutinous *sorghum* under Cd stress remain to be further studied.

In this study, we first performed transcriptome sequencing and metabolome detection on glutinous *sorghum* samples at 8 different stages after Cd treatment. After the identification of differentially expressed genes and differential metabolites, WGCNA association analysis was used to reveal the gene modules with strong correlation with differential metabolites, and the expression characteristics of the screened genes were verified by qRT-PCR. These studies will help us understand the genetic regulatory mechanisms and metabolite change characteristics of glutinous *sorghum* in response to Cd stress, and provide assistance for reducing Cd accumulation in *sorghum* and ensuring food safety.

## 2 Materials and methods

### 2.1 Plant materials

In this study, “HongYingZi”, a glutinous sorghum variety specifically used for liquor-making, was adopted as the experimental material. Its seeds were purchased from Guizhou Moutai Distillery (Group) Hongyingzi Agricultural Science and Technology Development Co., Ltd., and then cultivated through hydroponics in the climate chamber of the School of Food Engineering, Moutai University. After germination under hydroponic conditions, the seeds were further cultured for one week to obtain robust new shoots. To accurately simulate the nutrient environment required for the natural growth of plants, they were first cultured in Hoagland nutrient solution for another two weeks, after which control check (CK) samples were collected. It has been demonstrated in previous studies on sorghum roots that treatment with 150 μM Cd can induce abnormal growth of sorghum (Jiao et al., 2023). Considering that the above-ground parts of sorghum are less sensitive to Cd than the roots, 200 μM cadmium acetate was used for treatment in this study. Subsequently, corresponding samples were collected at 3 hours (TM3h), 6 hours (TM6h), 9 hours (TM9h), 12 hours (TM12h), 24 hours (TM24h), 48 hours (TM48h), and 72 hours (TM72h) post-treatment, respectively. The collected samples include all parts of the hydroponic seedlings except the roots, and each sample consists of three biological replicates.

### 2.2 RNA extraction and transcriptome sequencing

Total RNA was extracted from *sorghum* samples at 8 time points (CK, TM3h, TM6h, TM9h, TM12h, TM24h, TM48h, and TM72h) using a total RNA purification kit (cat. DP441, Tiangen Biochemical Technology, China) in accordance with the manufacturer’s protocol. The samples were then analyzed for RNA integrity and DNA contamination by agarose gel electrophoresis, and RNA concentration and integrity were detected using the NanoPhotometer spectrophotometer and the Agilent 2100 bioanalyzer, respectively. The qualified samples (CK, TM3h, TM6h, TM9h, TM12h, TM24h, TM48h, and TM72h) were further used for the construction of RNA library. Transcriptome sequencing was carried out by HiSeq 2500 system, and the reading length of sequencing was PE150. For more detailed methods, please refer to our previous research (An et al., 2024a).

### 2.3 Transcriptome data analysis

Before the transcriptome expression analysis, the raw reads are filtered by fastp software, and the filtering parameters are as follows: -length _ required = 25; -cut _ window _ size 4; -cut _ mean _ quality 15; -compression = 6 -w 50 (Chen et al., 2018). Subsequently, the reference genome of ‘HongYingZi’ *sorghum* was downloaded from http://sorghum.org.cn/#/downloads (Bao et al., 2024). Next, the filtered clean reads were aligned to the reference genome using Hisat2 software, and the resulting sam files from the alignment were converted to bam format and sorted using samtools software (Pertea et al., 2016). Transcript identification was performed using StringTie with default parameters, all newly generated gtf files were merged, and the gene expression abundance in *sorghum* samples under different treatments was estimated. And the R package DEGseq was used to identify differentially expressed genes (fold_change > 2 and the q-value < 0.05) (Pertea et al., 2016; An et al., 2025a). The transcription factors of *sorghum* genome were identified by iTAK (http://itak.feilab.net/cgi-bin/itak/index.cgi) (Zheng et al., 2016).

### 2.4 Untargeted metabolomics detection

All *sorghum* samples (CK, TM3h, TM6h, TM9h, TM12h, TM24h, TM48h, and TM72h) were placed in a freeze dryer (Scientz-100F) for vacuum freeze-drying for 63 hours, then ground into powder using a grinder (MM 400, Retsch) at 30 Hz for 1.5 minutes. 50 mg of the sample powder was weighed with an electronic balance (MS105DM), and 1200 μL of 70% methanol aqueous internal standard extract pre-cooled to -20°C was added (for samples less than 50 mg, the extractant was added at a ratio of 1200 μL per 50 mg of sample). The internal standard extract was prepared as follows: 1 mg of standard was dissolved in 1 mL of 70% methanol water to prepare a 1000 μg/mL standard stock solution, and the 1000 μg/mL stock solution was further diluted with 70% methanol to prepare a 250 μg/mL internal standard solution. Vortexing was performed once every 30 minutes, 30 seconds each time, for a total of 6 times. After centrifugation (12000 rpm for 3 minutes), the supernatant was aspirated, filtered through a microporous membrane (0.22 μm pore size), and stored in an injection vial for UPLC-MS/MS analysis. The chromatographic column used was Waters ACQUITY UPLC HSS T3 Column (1.8 µm, 2.1 mm × 100 mm). Mobile phase A: ultrapure water (containing 0.1% formic acid); mobile phase B: acetonitrile (containing 0.1% formic acid). Instrument column temperature: 40°C; flow rate: 0.40 mL/min; injection volume: 4 µL. The specific elution gradient is shown in **Supplementary Table S1**.

All the methods alternated between full scan MS and data dependent MSn scans using dynamic exclusion. MS analyses were carried out using electrospray ionization in the positive ion mode and negative ion mode using full scan analysis over m/z 84–1250 at 35000 resolution. Additional MS settings are: ion spray voltage, 3.5 KV or 3.2 KV in positive or negative modes, respectively; Sheath gas (Arb), 30; Aux gas, 5; Ion transfer tube temperature, 320°C; Vaporizer temperature, 300°C; Collision energy, 30,40,50 V; Signal Intensity Threshold, 1*e6 cps; Top N vs Top speed, 10; Exclusion duration, 3s. The raw data acquired from mass spectrometry (MS) was converted into mzML format using ProteoWizard. Subsequently, the XCMS program was employed for peak extraction, alignment, and retention time correction. Finally, substances with an integrated identification score of over 0.5 and a coefficient of variation (CV) value less than 0.5 in quality control (QC) samples were extracted. Further, positive and negative ion modes were merged (retaining the substances with the highest qualitative grade and the smallest CV value), resulting in the final detection data.

### 2.5 Identification of differential metabolites and series test of cluster

In order to identify the differential metabolites between different sample, Partial Least Squares Discriminant Analysis (PLS-DA) was performed based on the OmicShare platform (https://www.omicshare.com/) (Mu et al., 2024). The screening criteria of differential metabolites are VIP (Variable Importance in Projection) >1 and Pvalue <0.05 (An et al., 2025b). In addition, series test of cluster and KEGG enrichment analysis are also completed based on this platform.

### 2.6 Weighted gene co-expression network analysis

Based on the differentially expressed genes and untargeted metabolite data mentioned above, weighted gene co-expression network analysis (WGCNA) was performed using the WGCNA R package (Langfelder and Horvath, 2008; Qiao et al., 2021). The detailed parameters are set to: power = 17, maxBlockSize = 2000, TOMType = “unsigned”, minModuleSize = 50 and mergeCutHeight = 0.25. After gene clustering, different modules were identified, and metabolite content are correlated with each module.

### 2.7 Quantitative real-time PCR

Based on the results of differentially expressed genes and WGCNA, highly correlated genes were selected to evaluate their expression patterns in different *sorghum* treatment groups using qRT-PCR. The quality and quantity of each RNA extract was determined using agarose gel electrophoresis and Nanodrop 2500 (Thermo Fisher Scientific, US). Qualified RNA detected (The bands of 28S and 18S are clear, with OD260/OD230 ranging from 2.0-2.4 and OD260/OD280 ranging from 1.8-2.2) were used for subsequent experiments. The PrimeScript RT Kit (Cat. RR036A, Takara, Japan) was used to synthesize the first-strand cDNA following the manufacturer’s protocol. All cDNA were adjusted to around 150ng/ul using double distilled water. The Eukaryotic Initiation Factor 4A-1 (*eIF4α*) was used as the internal control gene (An et al., 2024b). Expression determination was performed with three biological replicates, and each replicate had three technical replicates. The relative gene expression values were analyzed using the 2^-△Ct^ method (Sudhakar Reddy et al., 2016). Detailed primer information is listed in **Supplementary Table S2**.

## 3 Results

### 3.1 Summary of transcriptome sequencing data

To reveal the overall expression profile of sorghum genes following Cd treatment, transcriptome sequencing was first performed on samples collected at a total of eight time points: CK, 3 hours, 6 hours, 9 hours, 12 hours, 24 hours, 48 hours, and 72 hours. The results of transcriptome sequencing data show that the amount of raw sequencing data for each biological replicate ranges from 5.94 Gb to 10.31 Gb. The clean reads obtained from the above data through strict filtering are between 5.93 and 10.29, and the average clean reads of each biological replicate reaches 7.32 Gb. Meanwhile, the average values of Q20, Q30, and GC_content reached 97.9%, 94.2%, and 51.6% respectively. Adequate sequencing data and good sequencing quality provide a solid foundation for the accurate analysis of gene expression levels (**Supplementary Table S3**).

### 3.2 Identification of differentially expressed genes and analysis of gene expression trends

**Figure 1:**
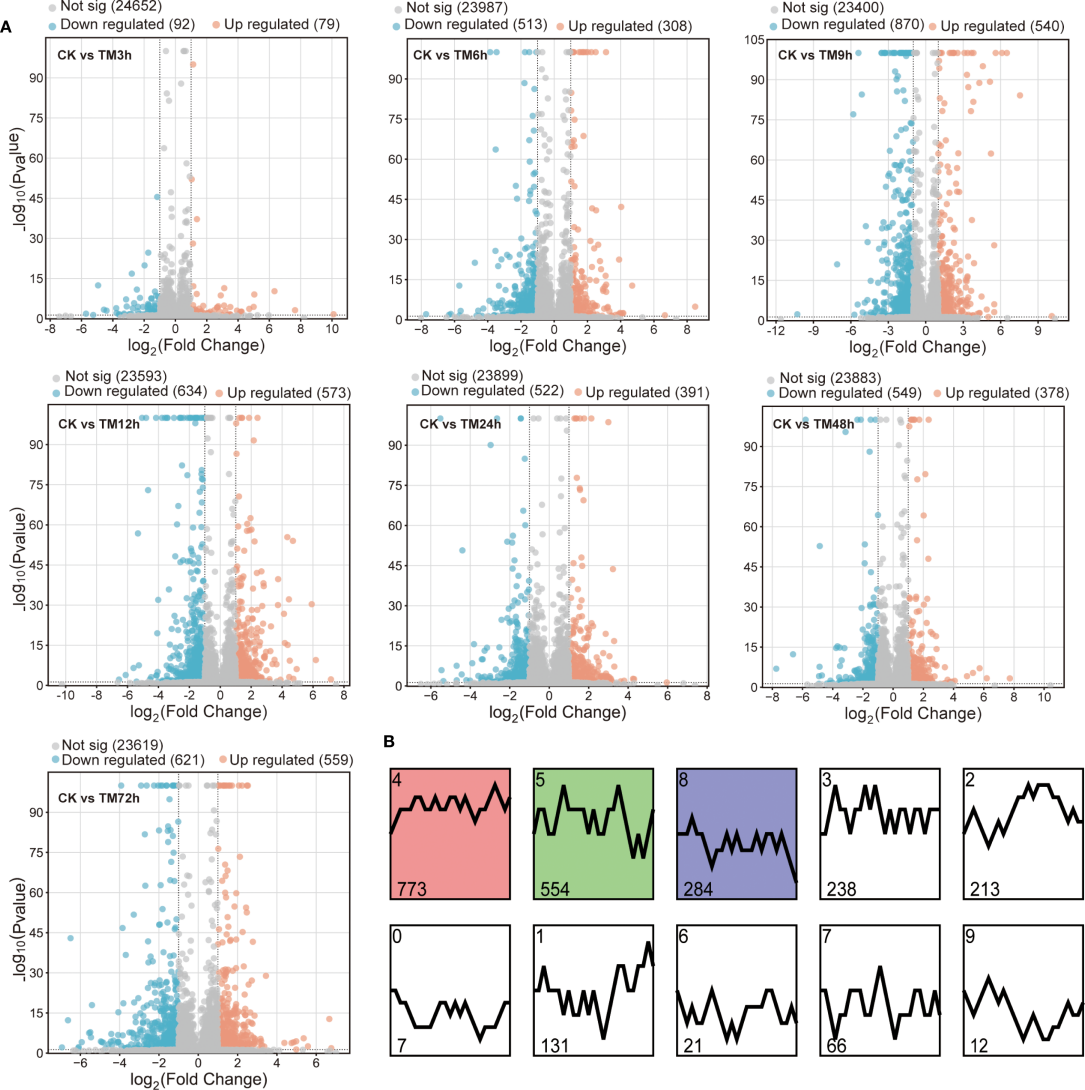
Expression patterns of *sorghum* genes after Cd treatment. **(A)** Differentially expressed genes in different treatment samples compared with CK; **(B)** Expression patterns of all differentially expressed genes, cluster 4, 5 and 8 were identified as significant trends, with p value <0.05.

After aligning the filtered clean reads to the reference genome to obtain the expression levels of all genes, we compared the samples of TM3h, TM6h, TM12h, TM24h, TM48h, and TM72h with the CK sample respectively to identify differentially expressed genes. As shown in **Figure 1A**, 92, 513, 870, 634, 522, 549, and 621 down-regulated genes were identified in CK vs TM3h, CK vs TM6h, CK vs TM9h, CK vs TM12h, CK vs TM24h, CK vs TM48h, and CK vs TM72h, respectively, while the number of up-regulated genes was 79, 308, 540, 573, 391, 378, and 559, respectively. Overall, the samples treated for 9 hours showed the largest number of differentially expressed genes compared to the CK samples, with the quantity reaching 1,410. Based on the KEGG enrichment analysis of differentially expressed genes (DEGs), it was found that more genes were enriched in pathways including circadian rhythm-plant, ATP-dependent chromatin remodeling, arachidonic acid metabolism, and starch and sucrose metabolism in the CK vs 24 and CK vs 48 comparison groups. In contrast, other groups were mainly significantly enriched in pathways such as Metabolic pathways, Anthocyanin biosynthesis, and Linoleic acid metabolism (**Supplementary Figure S1**). In all samples, a total of 2,299 differentially expressed genes were identified, which can be divided into 10 clusters according to different expression patterns (**Figure 1B**; **Supplementary Table S4**). Among them, cluster 4 contained the largest number of genes, reaching 773, while cluster 0 included only 7 genes. Meanwhile, clusters 4, 5, and 8 were identified as exhibiting significant expression trends. This indicates that many differentially expressed genes have similar expression patterns in different tissues.

### 3.3 Enrichment analysis of differential genes and identification of transcription factors

**Figure 2:**
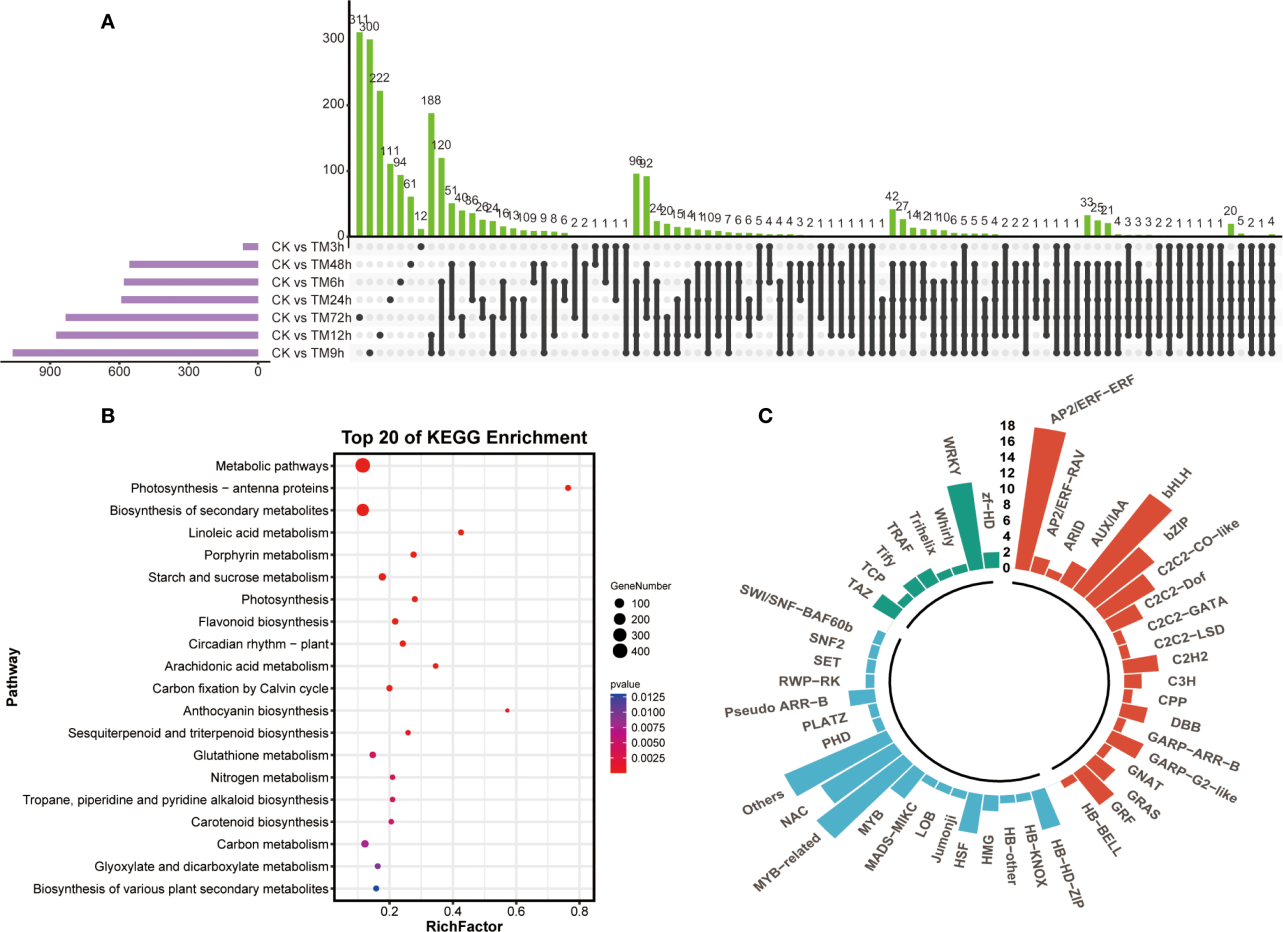
Comparison and enrichment analysis of differentially expressed genes. **(A)** Upset analysis of differentially expressed genes in different sample groups; **(B)** KEGG enrichment analysis of differentially expressed genes; **(C)** Identification of transcription factors identified in differentially expressed genes.

Next, we analyzed the unique differentially expressed genes among different comparison groups using an upset plot. As can be seen from **Figure 2A**, the three groups of CK vs TM72h, CK vs TM9h and CK vs TM12h contain the largest number of unique differentially expressed genes, with 311, 300 and 222 genes respectively. In addition, more unique intersections were found between CK vs TM6h - CK vs TM9h and CK vs TM9h - CK vs TM12h. The results of the KEGG enrichment analysis based on all differentially expressed genes indicated that a large number of differentially expressed genes were enriched in pathways such as metabolic pathways, biosynthesis of secondary metabolites, flavonoid biosynthesis, and starch and sucrose metabolism, suggesting that metabolites related to these pathways might be involved in the response of *sorghum* to Cd stress (**Figure 2B**). Meanwhile, among the 2,299 differentially expressed genes, a large number of transcription factors were identified, including many members of the WRKY, AP2/ERF, bHLH, and MYB-related families (**Figure 2C**).

### 3.4 Overview of metabolome data

**Figure 3:**
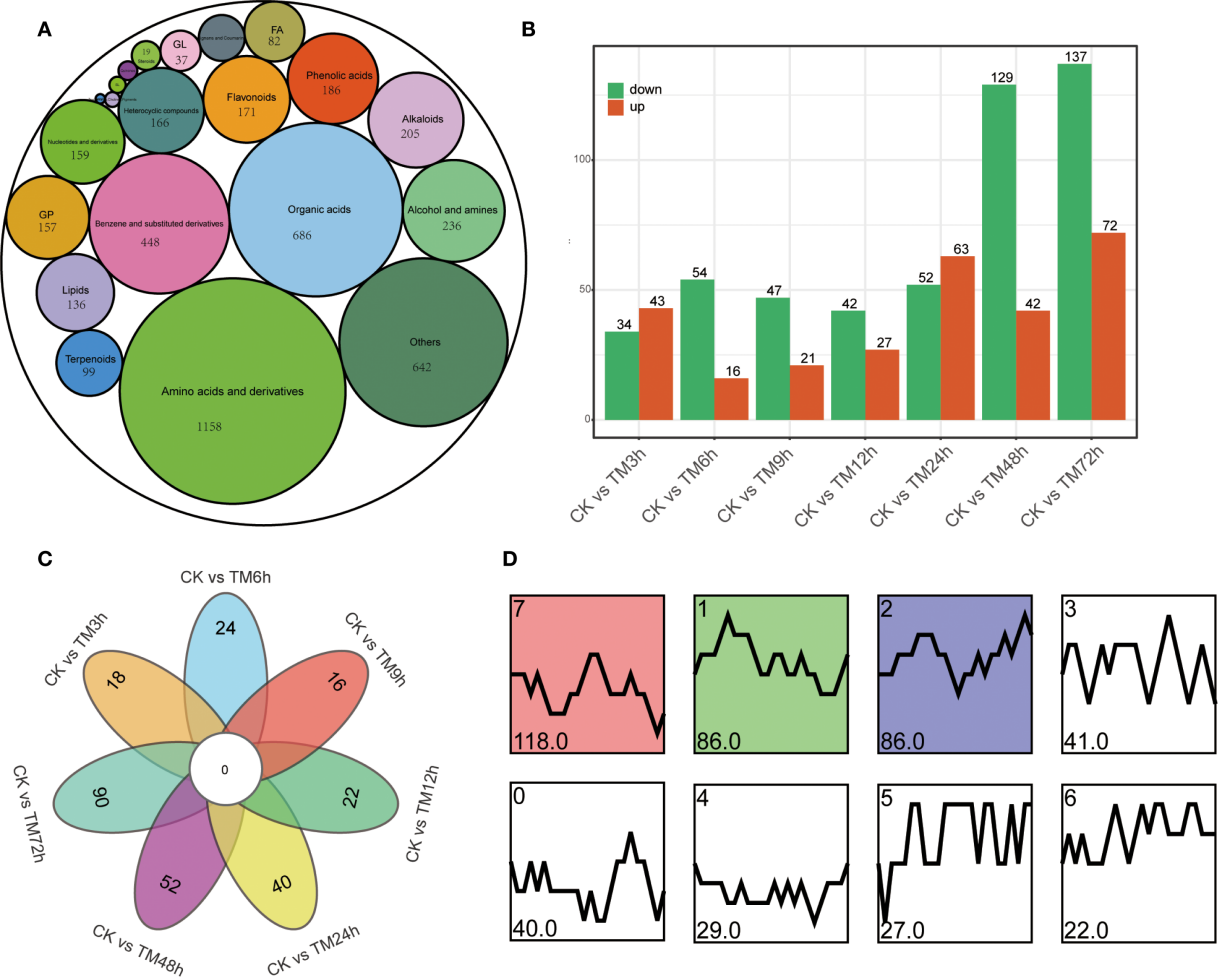
Type statistics and comparative analysis of differential metabolites. **(A)** Types of differential metabolites; **(B)** Differential metabolites between different treatment samples and CK; **(C)** Venn diagram showed specific differential metabolites in different comparison groups; **(D)** Change patterns of differential metabolites, cluster 1, 2 and 7 were identified as significant trends, with p value <0.05.

A total of 4,656 metabolites were identified through non-targeted metabolomics (**Supplementary Table S5**). These metabolites can be divided into 21 major categories, which mainly include 1,158 amino acids and their derivatives, 686 organic acids, 448 benzene and substituted derivatives, 236 alcohols and amines, 205 alkaloids, 171 flavonoids, and 99 terpenoids, among others (**Figure 3A**). A total of 77, 70, 68, 69, 115, 171, and 209 differential metabolites were identified in 7 groups, namely CK vs TM3h, CK vs TM6h, CK vs TM9h, CK vs TM12h, CK vs TM24h, CK vs TM48h, and CK vs TM72h, respectively (**Figure 3B**). As can be seen from **Figure 3C**, there is a relatively high proportion of unique differential metabolites among different control groups. For instance, in the comparisons of CK vs TM12h and CK vs TM72h, the proportions of unique differential metabolites are 34.8% and 43.1%, respectively. A total of 449 differential metabolites were identified in all samples (**Supplementary Table S6**). These metabolites were classified into 8 clusters based on their content characteristics. Among them, the cluster 1, 2 and 7 that exhibited significant trends contained the largest number of metabolites, with 86, 86, and 118 genes respectively. The number of differential metabolites contained in these three clusters alone accounts for 64.6% of the total differential metabolites.

### 3.5 Content characteristics of different types of metabolites

**Figure 4:**
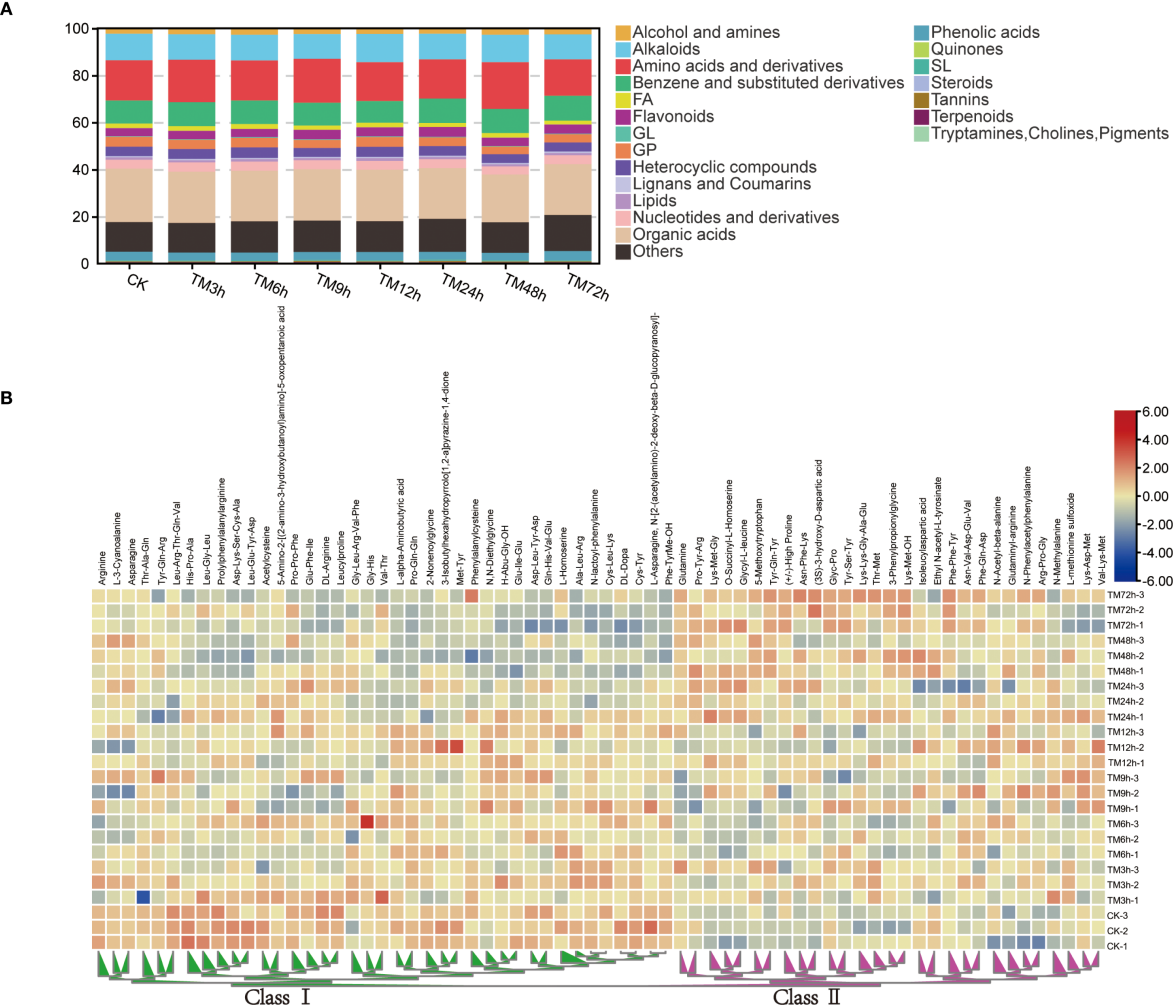
Content characteristics of different types of metabolites. **(A)** Proportion analysis of 21 types of metabolites in the total metabolites; **(B)** Heat map of the contents of differential amino acids and their derivatives, the two main branches of heat map are defined as Class I and Class II respectively.

Metabolomic analysis showed that there was no significant difference in the total metabolite content among different samples, and the overall difference in the content proportion of 21 metabolite classes across all treatments was relatively small. (**Supplementary Figure S2**). For example, the content of organic acids accounted for 20.3% to 22.7% of the total metabolites, while the proportion of amino acids and derivatives ranged from 15.5% to 19.9% (**Figure 4A**). Among all the differential metabolites, there are 16 flavonoids, 21 alkaloids, 92 organic acids, 23 phenolic acids, 43 benzene and substituted derivatives, 26 alcohols and amines, 68 amino acids and their derivatives, and other substances. Many studies have shown that changes in amino acid content are of great significance for plants to respond to Cd stress (Zhu et al., 2018; Jiao et al., 2023; Zhang et al., 2024). Therefore, we used a heatmap to display the content characteristics of all differential amino acids and their derivatives in different samples (**Figure 4B**). As can be seen from the figure, these differential metabolites can be generally divided into two categories. Overall, with the extension of Cd treatment time, the content of Class I amino acids shows a downward trend, while that of Class II shows the opposite trend.

### 3.6 WGCNA identifies genes related to amino acid metabolism

**Figure 5:**
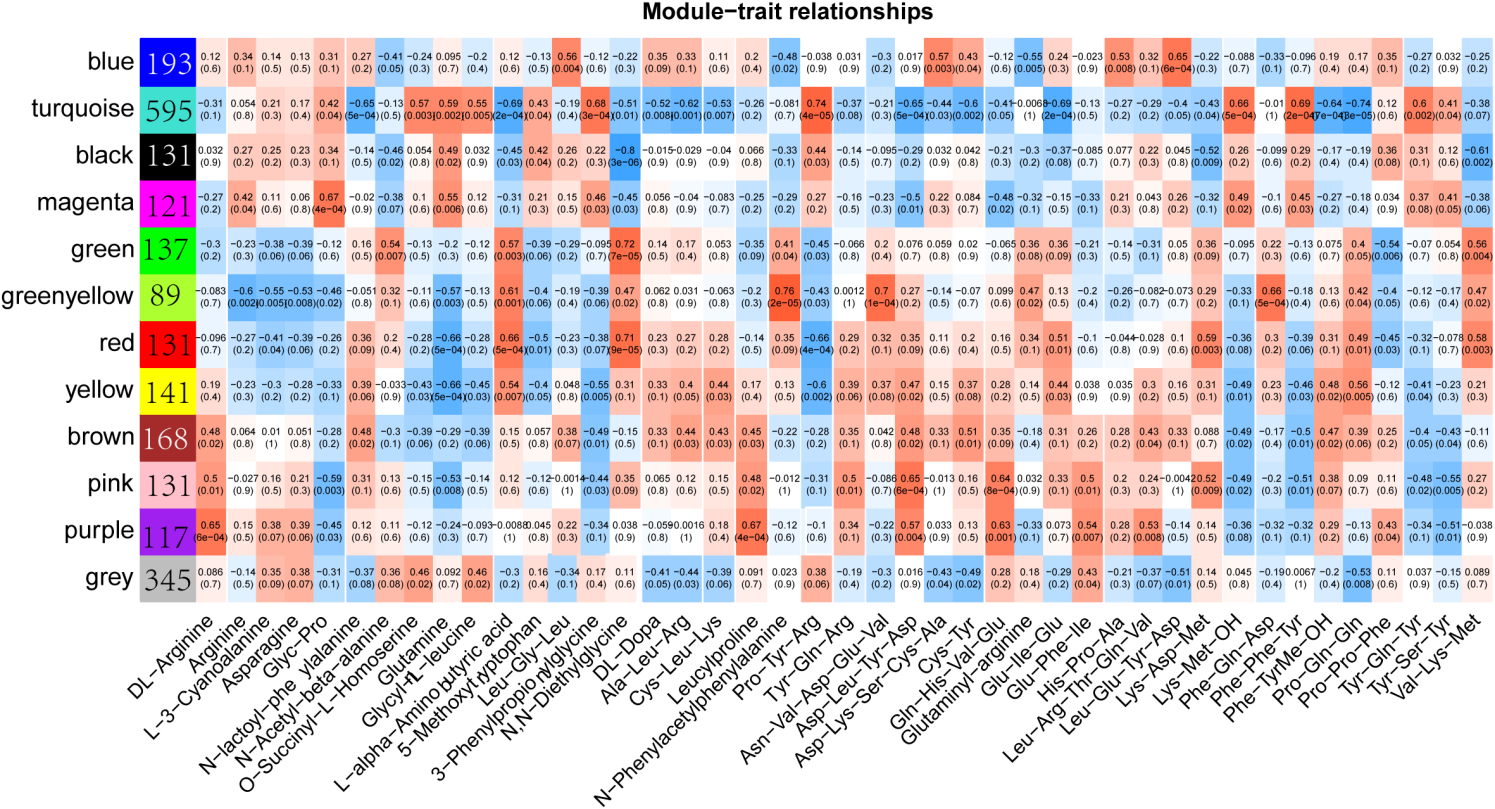
Weighted gene co-expression network analysis (WGCNA) of differentially expressed genes and differential amino acids and their derivatives. The color blocks on the left represent the clustering of related genes.

To investigate the relationship between the content of amino acids and their derivatives and differential genes, we performed a weighted gene co-expression network analysis using transcript expression levels and the content of 68 amino acids, which showed significant differences in different samples. The results showed that all differential genes were classified into 12 expression modules with different colors (**Figure 5**; **Supplementary Figure S3**). The number of genes in each module ranged from 89 (greenyellow module) to 595 (turquoise module). Each module was found to have a strong correlation with some specific amino acids. For example, the correlation between the gene in greenyellow module and N-Phenylacetylphenylalanine reached 0.76, and the correlation between the gene in turquoise module and Pro-Tyr-Arg reached 0.74. However, the genes in the black module show a strong negative correlation with N,N-diethylethanolamine, with the correlation coefficient reaching -0.8, while the genes in the green module have a positive correlation with N,N-Diethylglycine, with the correlation coefficient reaching 0.72. Overall, there are relatively few modules that are strongly correlated with amino acid contents, which indicates that there is a complex regulatory network for the amino acid contents in *sorghum* under Cd stress conditions.

**Figure 6:**
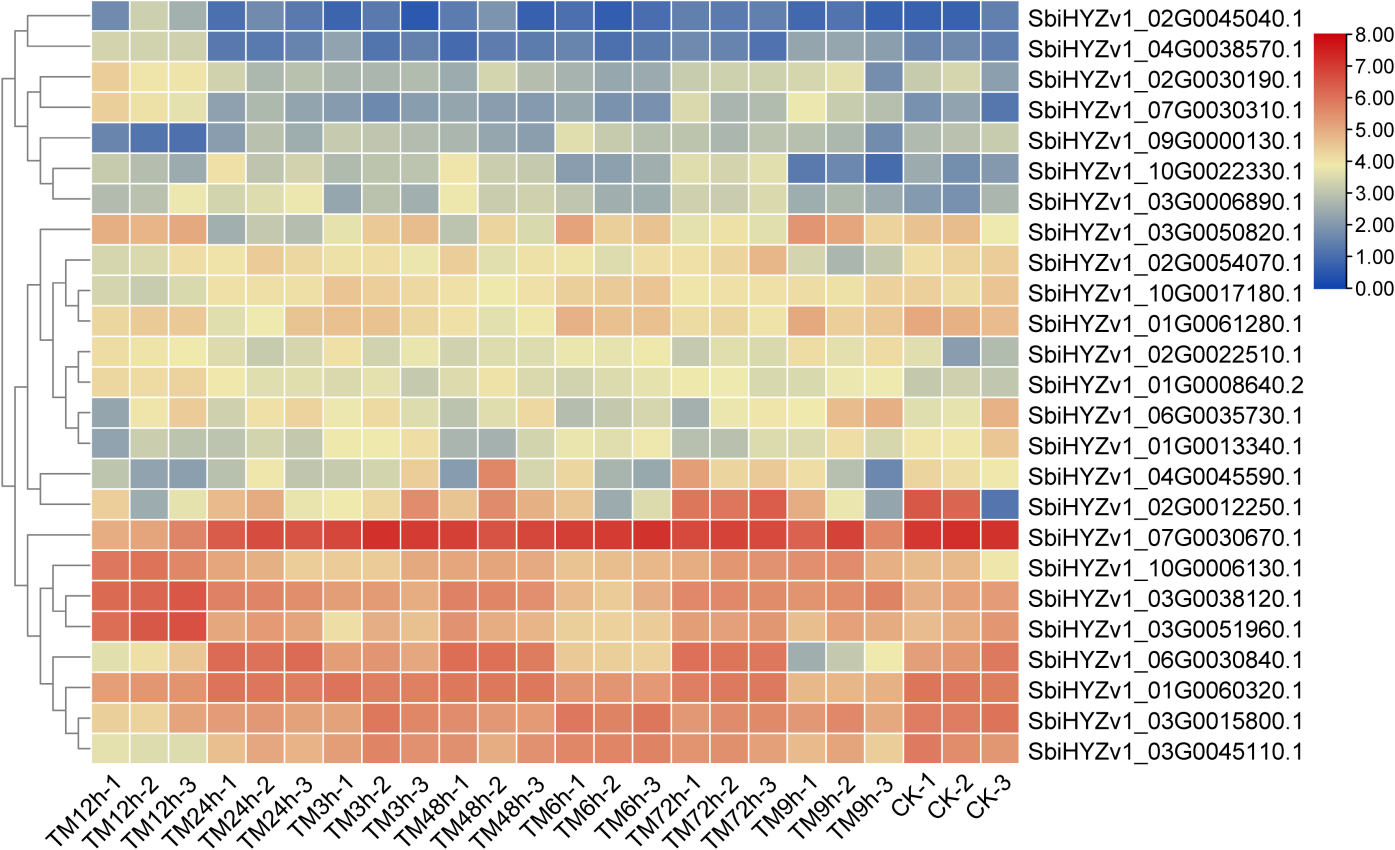
The heatmap shows the expression characteristics (FPKM) of some genes that have a high degree of correlation with the content of amino acids and their derivatives.

To reveal the expression characteristics of these genes, based on the results of WGCNA and correlation coefficient analysis, we selected some genes with a correlation coefficient greater than 0.64 and displayed their Fragments Per Kilobase of exon model per Million mapped fragments (FPKM) through a heatmap (**Figure 6**). The results showed that these genes may exhibit significant differences in different tissues. For example, the expression level of *SbiHYZv1_09G0000130.1* in TM12h was significantly lower, while *SbiHYZv1_02G0030190.1* was highly expressed in TM12h. Detailed gene expression data and functional descriptions have been listed in **Supplementary Table S4**.

### 3.7 Verification of gene expression levels by qRT-PCR

**Figure 7:**
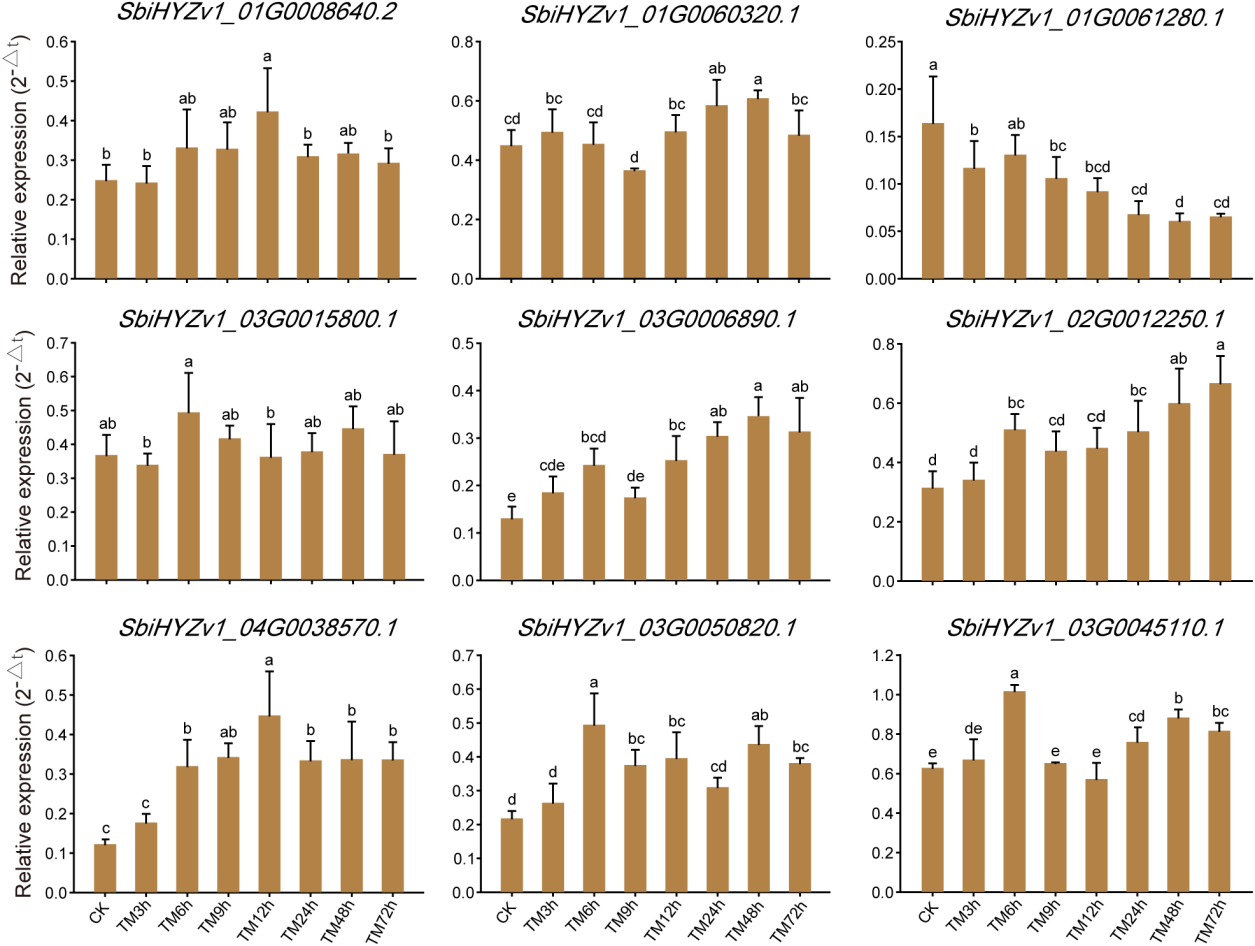
Quantitatively verify the expression levels of genes with a high correlation to amino acid content through qRT-PCR. Different letter combinations on the bars in the figure indicate significant differences. When the letters above any two columns are completely different, it indicates that there is a significant difference in the gene expression levels between these two groups (P<0.05).

To further verify the reliability of the transcriptome data and the actual expression levels of genes in the relevant modules, we randomly selected 9 representative genes for qRT-PCR validation. As shown in **Figure 7** and **Supplementary Table S7**, *SbiHYZv101G0008640.2* had the highest expression level in TM12h, *SbiHYZv101G0061280.1* had the highest expression level in CK, while *SbiHYZv102G0012250.1* showed the highest expression level in the 72h sample. These results are highly consistent with the transcriptome data, further indicating the reliability of our results. Meanwhile, these genes were annotated as Thioredoxin domain-containing protein (*SbiHYZv1_01G0008640.2*), HMA domain-containing protein (*SbiHYZv1_01G0060320.1*), Alba domain-containing protein (*SbiHYZv1_01G0061280.1*), LRRNT_2 domain-containing protein (*SbiHYZv1_03G0006890.1*), MFS domain-containing protein (*SbiHYZv1_03G0015800.1*), Epimerase domain-containing protein (*SbiHYZv1_03G0045110.1*), S1 motif domain-containing protein (*SbiHYZv1_03G0050820.1*), and PPM-type phosphatase domain-containing protein (*SbiHYZv1_04G0038570.1*).

## 4 Discussion

Cd is the most serious major metal pollutant in farmland soil (Wang et al., 2019). The Cd element absorbed by plants through their roots not only affects the growth, development, and compound synthesis of plants, but can also be ingested by humans through the food chain, posing a potential food safety risk (Liu et al., 2020; Xing et al., 2025). Studies have shown that most gramineous plants have a strong ability to absorb Cd, which can cause the Cd content in grains to exceed the limit standards. Although this has certain significance for the remediation of Cd-contaminated soils, the Cd retained during processing may lead to the risk of excessive exposure among the population (Zou et al., 2021). In sweet *sorghum*, extensive research has been conducted on Cd stress and accumulation. For instance, it was found that yellow stripe-like genes (*SbYS1*) and WRKY domain-containing protein gene family (*SbWRKY72)* in *sorghum* synergistically regulate cadmium absorption (Jia et al., 2024); transcriptome analysis showed that zinc transporter 9 (*SbZIP9*) and flavonoid O-methyltransferase-like gene (*SbFOMT-like*) enhance and reduce Cd accumulation respectively under the regulation of potassium addition (Zhang et al., 2024). However, the genetic regulatory mechanisms and changes in metabolites of glutinous *sorghum* (brewing *sorghum*) after Cd treatment have not yet been clearly revealed.

To reveal the genetic mechanisms and characteristics of metabolite changes in glutinous *sorghum* in response to Cd treatment, we selected a total of eight groups of samples at different periods, including the CK samples, for transcriptome sequencing and metabolome analysis after subjecting the brewing glutinous *sorghum* variety ‘HongYingZi’ to Cd treatment. Ultimately, we obtained a total of 176.2 Gb of raw sequencing data, with an average of 7.32 Gb of clean data per sample and a Q30 value of over 94% (**Supplementary Table S3**). The sufficient high-quality sequencing data provides a foundation for further identification of differential genes. Compared with CK, the TM9h samples had the largest number of differentially expressed genes identified, while the TM3h samples showed little difference from CK. A total of 2,299 differential genes were identified across all samples. These genes were classified into 10 categories based on their expression patterns, among which the genes in cluster 4 and cluster 5 accounted for 57.7% in total (**Figure 1**). This indicates that most differential genes exhibit similar expression patterns after Cd treatment. The results of KEGG enrichment analysis showed that these genes were mainly enriched in metabolic pathways, biosynthesis of secondary metabolites, and flavonoid biosynthesis (**Figure 2B**). Meanwhile, these differentially expressed genes include many important transcription factors such as AP2/ERF, bHLH, NAC, and MYB-related, which may play a role in resisting abiotic stresses like Cd (**Figure 2C**). For example, *MYB49* can directly bind to the promoters of basic helix-loop-helix transcription factors bHLH38 and *bHLH101*, positively regulate their expression, leading to the activation of IRON-*REGULATED TRANSPORTER1*, and thus participate in the uptake and transport of Cd (Zhang et al., 2019). Studies in *Arabidopsis thaliana* have found that the transcription factor *NAC102* confers Cd tolerance through the expression of *WAKL11* and cell wall pectin metabolism (Han et al., 2023).

Both biotic and abiotic stresses can affect the metabolic characteristics of plants to varying degrees (Wang et al., 2016; Zhao et al., 2020). In this study, we identified a total of 4,656 metabolites, which were classified into 21 major categories. These mainly include 1158 amino acids and their derivatives, 686 organic acids, 448 benzene and substituted derivatives, 236 alcohols and amines, 205 alkaloids, and 171 flavonoids, among others (**Figure 3A**). In general, with the increase of processing time, the differential metabolites between the treated samples and the CK samples showed a gradual increase trend (**Figure 3B**). However, there is no significant difference in the total content of metabolites among different samples (**Figure 4A**). Meanwhile, a total of 449 differential metabolites were identified, among which amino acids and their derivatives were the most abundant, accounting for 68 (**Figure 4B**). Numerous studies have shown that Cd stress can affect amino acid metabolism, so we focused on the regulatory mechanisms of these differential amino acids (Zemanová et al., 2017; Zhu et al., 2018; Li et al., 2024a). Through WGCNA correlation analysis, we identified 12 gene modules related to the metabolism of these amino acids. Each module contains a varying number of genes, ranging from 89 to 595, including HMA domain-containing protein, PPM-type phosphatase domain-containing protein, and Thioredoxin domain-containing protein, among others (**Figure 5**). These genes are directly or indirectly involved in the response to Cd stress and the regulation of amino acids (Liao et al., 2023; Kamada et al., 2020). Each module shows a high correlation with at least one or more amino acids, suggesting that these amino acids have complex regulatory mechanisms in *sorghum*.

In addition, based on the transcriptome analysis, we also randomly selected genes with high correlation to verify their expression levels using qRT-PCR. The results showed that the expression levels of these genes were highly consistent with the transcriptome results (**Figure 7**). Our study comprehensively demonstrates the gene expression profiles and metabolite change characteristics of *sorghum* under Cd treatment conditions, providing a basis for further in-depth research on its genetic mechanisms.

## 5 Conclusion

In this study, based on transcriptome sequencing and metabolomics detection, the gene expression profile and metabolite changes of sorghum under cadmium treatment were analyzed. A total of 2299 differentially expressed genes were identified, and these genes were mainly enriched into metabolic pathways, biosynthesis of secondary metabolites and other pathways. Among the 449 differential metabolites, the most variable belonged to amino acids and their derivatives, and 12 gene modules were identified as related to their metabolism through WGCNA. These research findings reveal the regulatory basis and metabolic flux changes of sorghum in response to Cd stress, and provide a foundation and support for further research on functional genes related to metabolic synthesis or stress response.

## Data Availability

The datasets presented in this study can be found in online repositories. The names of the repository/repositories and accession number(s) can be found below: https://ngdc.cncb.ac.cn/gsa, CRA028452.

## Supplementary material

Supplementary figure S1 KEGG enrichment analysis of differentially expressed genes. A, B, C, D, E, F, and G represent CK vs TM3h, CK vs TM6h, CK vs TM9h, CK vs TM12h, CK vs TM24h, CK vs TM48h, and CK vs TM72h, respectively.

Supplementary figure S2 Relative contents of metabolites in samples at different time points.

Supplementary figure S3 Gene clustering tree obtained from WGCNA analysis

## References

[B1] An Y. Li X. Chen Y. Jiang S. Jing T. Zhang F. (2024a). Genome-wide identification of the OVATE gene family and revelation of its expression profile and functional role in eight tissues of rosa roxburghii tratt. BMC Plant Biol. 24, 1068. doi: 10.1186/s12870-024-05775-1, PMID: 39538133 PMC11558829

[B2] An Y. Wu J. Chen Y. Li S. (2025a). Comprehensive analysis of alternative splicing in rosa roxburghii tratt reveals its role in flavonoid synthesis. Front. Plant Sci. 16. doi: 10.3389/fpls.2025.1627126, PMID: 40718022 PMC12291298

[B3] An Y. Xia X. Zhang X. Liu L. Jiang S. Jing T.. (2024b). Genome-wide identification of the sorghum OVATE gene family and revelation of its expression characteristics in sorghum seeds and leaves. Sci. Rep. 14, 15123. doi: 10.1038/s41598-024-66103-z, PMID: 38956272 PMC11219837

[B4] An Y. Zhang L. Li X. Mi X. Qiao D. Jing T. (2025b). Integrated multi-omics reveals distinct non-volatile and aroma signatures in albino, yellow, and purple tea varieties. Food Chem.: X 29, 102830. doi: 10.1016/j.fochx.2025.102830, PMID: 40761751 PMC12320703

[B5] Bao J. Zhang H. Wang F. Li L. Zhu X. Xu J.. (2024). Telomere-to-telomere genome assemblies of two chinese baijiu-brewing sorghum landraces. Plant Commun. 5(6), 100933. doi: 10.1016/j.xplc.2024.100933, PMID: 38689492 PMC11211619

[B6] Butaciu S. Frentiu T. Senila M. Darvasi E. Cadar S. Ponta M.. (2016). Determination of cd in food using an electrothermal vaporization capacitively coupled plasma microtorch optical emission microspectrometer: compliance with european legislation and comparison with graphite furnace atomic absorption spectrometry. Food Control 61, 227–234. doi: 10.1016/j.foodcont.2015.09.040

[B7] Chen S. Zhou Y. Chen Y. Gu J. (2018). fastp: An ultra-fast all-in-one FASTQ preprocessor. Bioinformatics 34, i884–i890. doi: 10.1093/bioinformatics/bty560, PMID: 30423086 PMC6129281

[B8] Feng J. Jia W. Lv S. Bao H. Miao F. Zhang X.. (2018). Comparative transcriptome combined with morpho-physiological analyses revealed key factors for differential cadmium accumulation in two contrasting sweet sorghum genotypes. Plant Biotechnol. J. 16, 558–571. doi: 10.1111/pbi.12795, PMID: 28703450 PMC5787832

[B9] Gladman N. Olson A. Wei S. Chougule K. Lu Z. Tello-Ruiz M.. (2022). SorghumBase: A web-based portal for sorghum genetic information and community advancement. Planta 255, 35. doi: 10.1007/s00425-022-03821-6, PMID: 35015132 PMC8752523

[B10] Guo J. Chi J. (2014). Effect of cd-tolerant plant growth-promoting rhizobium on plant growth and cd uptake by lolium multiflorum lam. and glycine max (L.) merr. in cd-contaminated soil. Plant Soil 375, 205–214. doi: 10.1007/s11104-013-1952-1

[B11] Haider F. U. Liqun C. Coulter J. A. Cheema S. A. Wu J. Zhang R.. (2021). Cadmium toxicity in plants: impacts and remediation strategies. Ecotoxicol. Environ. Saf. 211, 111887. doi: 10.1016/j.ecoenv.2020.111887, PMID: 33450535

[B12] Han G. H. Huang R. N. Hong L. H. Xu J. X. Hong Y. G. Wu Y. H.. (2023). The transcription factor NAC102 confers cadmium tolerance by regulating *WAKL11* expression and cell wall pectin metabolism in *arabidopsis* . J. Integr. Plant Biol. 65, 2262–2278. doi: 10.1111/jipb.13557, PMID: 37565550

[B13] Jia W. Guo Z. Lv S. Lin K. Li Y. (2024). SbYS1 and SbWRKY72 regulate cd tolerance and accumulation in sweet sorghum. Planta 259, 100. doi: 10.1007/s00425-024-04388-0, PMID: 38536457

[B14] Jia W. Miao F. Lv S. Feng J. Zhou S. Zhang X.. (2017). Identification for the capability of cd-tolerance, accumulation and translocation of 96 sorghum genotypes. Ecotoxicol. Environ. Saf. 145, 391–397. doi: 10.1016/j.ecoenv.2017.07.002, PMID: 28759768

[B15] Jiao Z. Shi Y. Wang J. Wang Z. Zhang X. Jia X.. (2023). Integration of transcriptome and metabolome analyses reveals sorghum roots responding to cadmium stress through regulation of the flavonoid biosynthesis pathway. Front. Plant Sci. 14. doi: 10.3389/fpls.2023.1144265, PMID: 36909379 PMC9996021

[B16] Jing H. Yang W. Chen Y. Yang L. Zhou H. Yang Y.. (2023). Exploring the mechanism of cd uptake and translocation in rice: future perspectives of rice safety. Sci Total Environ. 897, 165369. doi: 10.1016/j.scitotenv.2023.165369, PMID: 37433335

[B17] Kamada R Kudoh F. Ito S. Tani I. Janairo J. I.B. Omichinski J. G.. (2020). Metal-dependent Ser/Thr protein phosphatase PPM family: Evolution, structures, diseases and inhibitors. Pharmacology & Therapeutics. 215, 107622. doi: 10.1016/j.pharmthera.2020.107622, PMID: 32650009

[B18] Langfelder P. Horvath S. (2008). WGCNA: an R package for weighted correlation network analysis. BMC Bioinf. 9, 559. doi: 10.1186/1471-2105-9-559, PMID: 19114008 PMC2631488

[B19] Li H. Luo N. Li Y. W. Cai Q. Y. Li H. Y. Mo C. H.. (2017). Cadmium in rice: transport mechanisms, influencing factors, and minimizing measures. Environ. pollut. 224, 622–630. doi: 10.1016/j.envpol.2017.01.087, PMID: 28242254

[B20] Li L. Chen Q. Cui S. Ishfaq M. Zhou L. Zhou X.. (2024a). Exogenous application of amino acids alleviates toxicity in two chinese cabbage cultivars by modulating cadmium distribution and reducing its translocation. Int. J. Mol. Sci. 25, 8478. doi: 10.3390/ijms25158478, PMID: 39126047 PMC11313598

[B21] Li Q. Wang J. Liu Q. Zhang J. Zhu X. Hua Y.. (2024b). Revealing critical mechanisms in determining sorghum resistance to drought and salt using mRNA, small RNA and degradome sequencing. BMC Plant Biol. 24, 547. doi: 10.1186/s12870-024-05230-1, PMID: 38872092 PMC11177356

[B22] Liao C. Li Y. Wu X. Wu W. Zhang Y. Zhan P.. (2023). ZmHMA3, a member of the heavy-metal-transporting ATPase family, regulates cd and zn tolerance in maize. Int. J. Mol. Sci. 24, 13496. doi: 10.3390/ijms241713496, PMID: 37686302 PMC10487686

[B23] Liu M. Liu X. Kang J. Korpelainen H. Li C. (2020). Are males and females of populus cathayana differentially sensitive to cd stress? J. Hazard. Mater. 393, 122411. doi: 10.1016/j.jhazmat.2020.122411, PMID: 32114141

[B24] Mu H. Chen J. Huang W. Huang G. Deng M. Hong S.. (2024). OmicShare tools: A zero-code interactive online platform for biological data analysis and visualization. iMeta 3, e228. doi: 10.1002/imt2.228, PMID: 39429881 PMC11488081

[B25] Nguyen C. Soulier A. J. Masson P. Bussière S. Cornu J. Y. (2016). Accumulation of cd, cu and zn in shoots of maize (zea mays L.) exposed to 0.8 or 20 nM cd during vegetative growth and the relation with xylem sap composition. Environ. Sci. pollut. Res. 23, 3152–3164. doi: 10.1007/s11356-015-5782-y, PMID: 26573313

[B26] Pertea M. Kim D. Pertea G. M. Leek J. T. Salzberg S. L. (2016). Transcript-level expression analysis of RNA-seq experiments with HISAT, StringTie and ballgown. Nat. Protoc. 11, 1650–1667. doi: 10.1038/nprot.2016.095, PMID: 27560171 PMC5032908

[B27] Qiao D. Mi X. An Y. Xie H. Cao K. Chen H.. (2021). Integrated metabolic phenotypes and gene expression profiles revealed the effect of spreading on aroma volatiles formation in postharvest leaves of green tea. Food Res. Int. 149, 110680. doi: 10.1016/j.foodres.2021.110680, PMID: 34600682

[B28] Rasin P. Ashwanti A. V. Basheer S. M. Haribabu J. Santibanez J. F. Garrote C. A.. (2025). Exposure to cadmium and its impacts on human health: A short review. J. Hazardous Materials Adv. 17, 100608. doi: 10.1016/j.hazadv.2025.100608

[B29] Shan X. Dou F. Li D. Yuan Y. Zhang Y. Liu C. (2025). Cadmium accumulation and translocation in maize cultivars on contaminated soils in southern China. BMC Plant Biol. 25, 589. doi: 10.1186/s12870-025-06286-3, PMID: 40325406 PMC12051276

[B30] Sudhakar Reddy P. Srinivas Reddy D. Sivasakthi K. Bhatnagar-Mathur P. Vadez V. Sharma K. K. (2016). Evaluation of sorghum [sorghum bicolor (L.)] reference genes in various tissues and under abiotic stress conditions for quantitative real-time PCR data normalization. Front. Plant Sci. 7. doi: 10.3389/fpls.2016.00529, PMID: 27200008 PMC4843019

[B31] Tao J. Lu L. (2022). Advances in genes-encoding transporters for cadmium uptake, translocation, and accumulation in plants. Toxics 10, 411. doi: 10.3390/toxics10080411, PMID: 35893843 PMC9332107

[B32] Tao Y. Luo H. Xu J. Cruickshank A. Zhao X. Teng F.. (2021). Extensive variation within the pan-genome of cultivated and wild sorghum. Nat. Plants 7, 766–773. doi: 10.1038/s41477-021-00925-x, PMID: 34017083

[B33] Wang P. Chen H. Kopittke P. M. Zhao F.-J. (2019). Cadmium contamination in agricultural soils of China and the impact on food safety. Environ. pollut. 249, 1038–1048. doi: 10.1016/j.envpol.2019.03.063, PMID: 31146310

[B34] Wang L. Cui X. Cheng H. Chen F. Wang J. Zhao X.. (2015). A review of soil cadmium contamination in China including a health risk assessment. Environ. Sci. pollut. Res. 22, 16441–16452. doi: 10.1007/s11356-015-5273-1, PMID: 26362640

[B35] Wang Y.-N. Tang L. Hou Y. Wang P. Yang H. Wei C.-L. (2016). Differential transcriptome analysis of leaves of tea plant (camellia sinensis) provides comprehensive insights into the defense responses to ectropis oblique attack using RNA-seq. Funct. Integr. Genomics 16, 383–398. doi: 10.1007/s10142-016-0491-2, PMID: 27098524

[B36] Wei C. Gao L. Xiao R. Wang Y. Chen B. Zou W.. (2024). Complete telomere-to-telomere assemblies of two sorghum genomes to guide biological discovery. Imeta 3, e193. doi: 10.1002/imt2.193, PMID: 38882488 PMC11170960

[B37] Xing A. Dai L. Zhang Y. Sui X. Wu Z. Wan S.. (2025). Application of exogenous hydrogen sulfide alleviates cadmium-induced stress on tea plant growth. Plant Stress 17, 100968. doi: 10.1016/j.stress.2025.100968

[B38] Zemanová V. Pavlík M. Pavlíková D. (2017). Cadmium toxicity induced contrasting patterns of concentrations of free sarcosine, specific amino acids and selected microelements in two noccaea species. PloS One 12, e0177963. doi: 10.1371/journal.pone.0177963, PMID: 28542385 PMC5438182

[B39] Zhang P. Li J. Li T. Li X. Lu Y. Wu J. (2024). Transcriptome analysis of potassium-mediated cadmium accumulation in sweet sorghum. Plant Physiol. Biochem. 215, 109064. doi: 10.1016/j.plaphy.2024.109064, PMID: 39191042

[B40] Zhang P. Wang R. Ju Q. Li W. Tran L.-S. P. Xu J. (2019). The R2R3-MYB transcription factor MYB49 regulates cadmium accumulation. Plant Physiol. 180, 529–542. doi: 10.1104/pp.18.01380, PMID: 30782964 PMC6501104

[B41] Zhao X. Luo L. Cao Y. Liu Y. Li Y. Wu W.. (2018). Genome-wide association analysis and QTL mapping reveal the genetic control of cadmium accumulation in maize leaf. BMC Genomics 19, 91. doi: 10.1186/s12864-017-4395-x, PMID: 29370753 PMC5785805

[B42] Zhao M. Zhang N. Gao T. Jin J. Jing T. Wang J.. (2020). Sesquiterpene glucosylation mediated by glucosyltransferase UGT91Q2 is involved in the modulation of cold stress tolerance in tea plants. New Phytol. 226, 362–372. doi: 10.1111/nph.16364, PMID: 31828806

[B43] Zheng H. Dang Y. Diao X. Sui N. (2024). Molecular mechanisms of stress resistance in sorghum: implications for crop improvement strategies. J. Integr. Agric. 23, 741–768. doi: 10.1016/j.jia.2023.12.023

[B44] Zheng H. Gao Y. Sui Y. Dang Y. Wu F. Wang X.. (2023). R2R3 MYB transcription factor SbMYBHv33 negatively regulates sorghum biomass accumulation and salt tolerance. Theor. Appl. Genet. 136, 5. doi: 10.1007/s00122-023-04292-3, PMID: 36656365

[B45] Zheng Y. Jiao C. Sun H. Rosli H. G. Pombo M. A. Zhang P.. (2016). iTAK: A program for genome-wide prediction and classification of plant transcription factors, transcriptional regulators, and protein kinases. Mol. Plant 9, 1667–1670. doi: 10.1016/j.molp.2016.09.014, PMID: 27717919

[B46] Zhu G. Xiao H. Guo Q. Zhang Z. Zhao J. Yang D. (2018). Effects of cadmium stress on growth and amino acid metabolism in two compositae plants. Ecotoxicology Environ. Saf. 158, 300–308. doi: 10.1016/j.ecoenv.2018.04.045, PMID: 29727812

[B47] Zou M. Zhou S. Zhou Y. Jia Z. Guo T. Wang J. (2021). Cadmium pollution of soil-rice ecosystems in rice cultivation dominated regions in China: a review. Environ. pollut. 280, 116965. doi: 10.1016/j.envpol.2021.116965, PMID: 33774546

