# Supplementary figures and images for: Integrating transcriptome and metabolome to reveal the characteristics of gene expression profiles and metabolite changes of sorghum in response to cadmium treatment
Source: Front Plant Sci. 2025 Sep 18;16:1678876. doi: 10.3389/fpls.2025.1678876 (PMC12488672; doi:10.3389/fpls.2025.1678876)

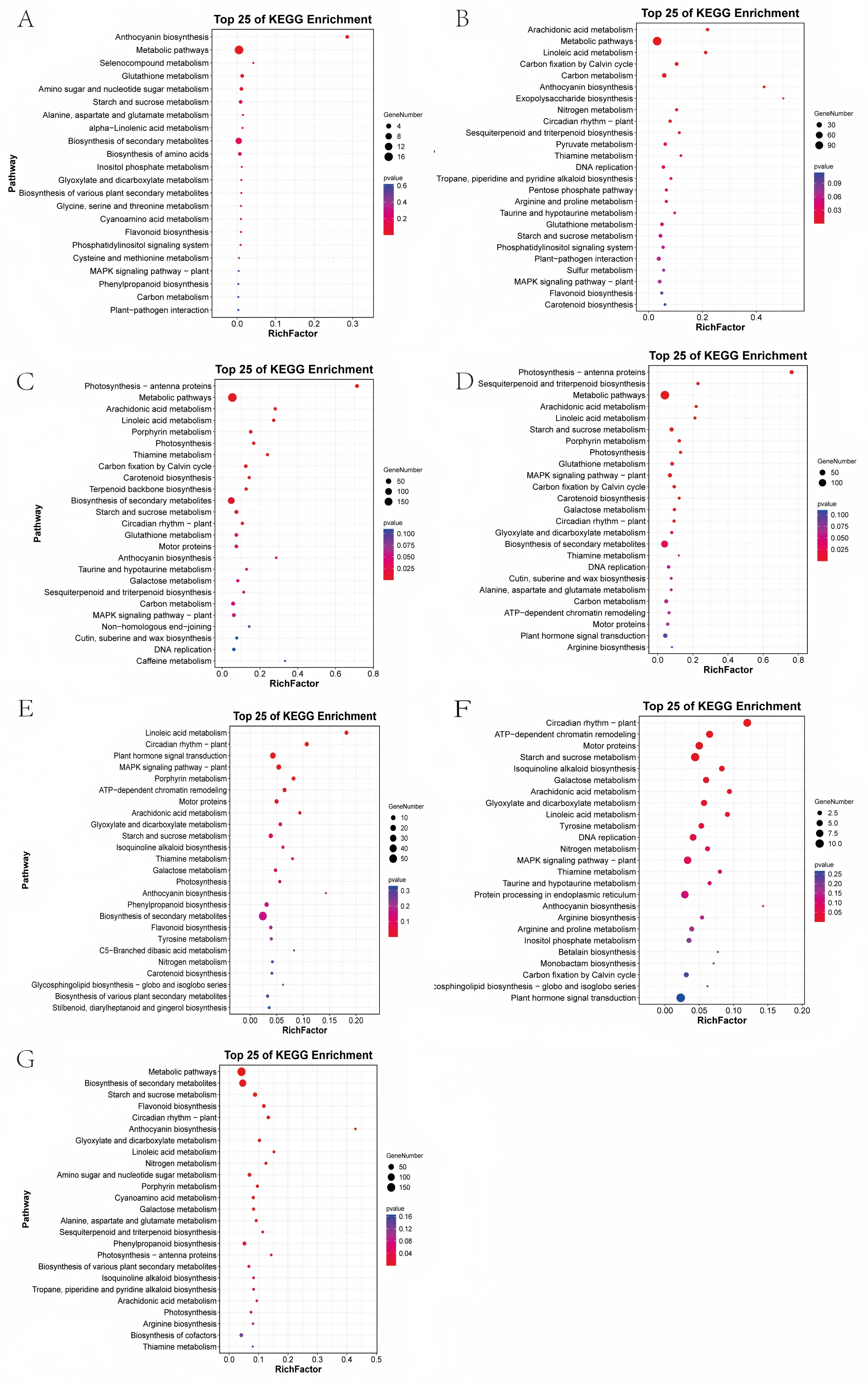

Supplement: Supplementary figure S1 — KEGG enrichment analysis of differentially expressed genes. A, B, C, D, E, F, and G represent CK vs TM3h, CK vs TM6h, CK vs TM9h, CK vs TM12h, CK vs TM24h, CK vs TM48h, and CK vs TM72h, respectively. [file Image1.png]

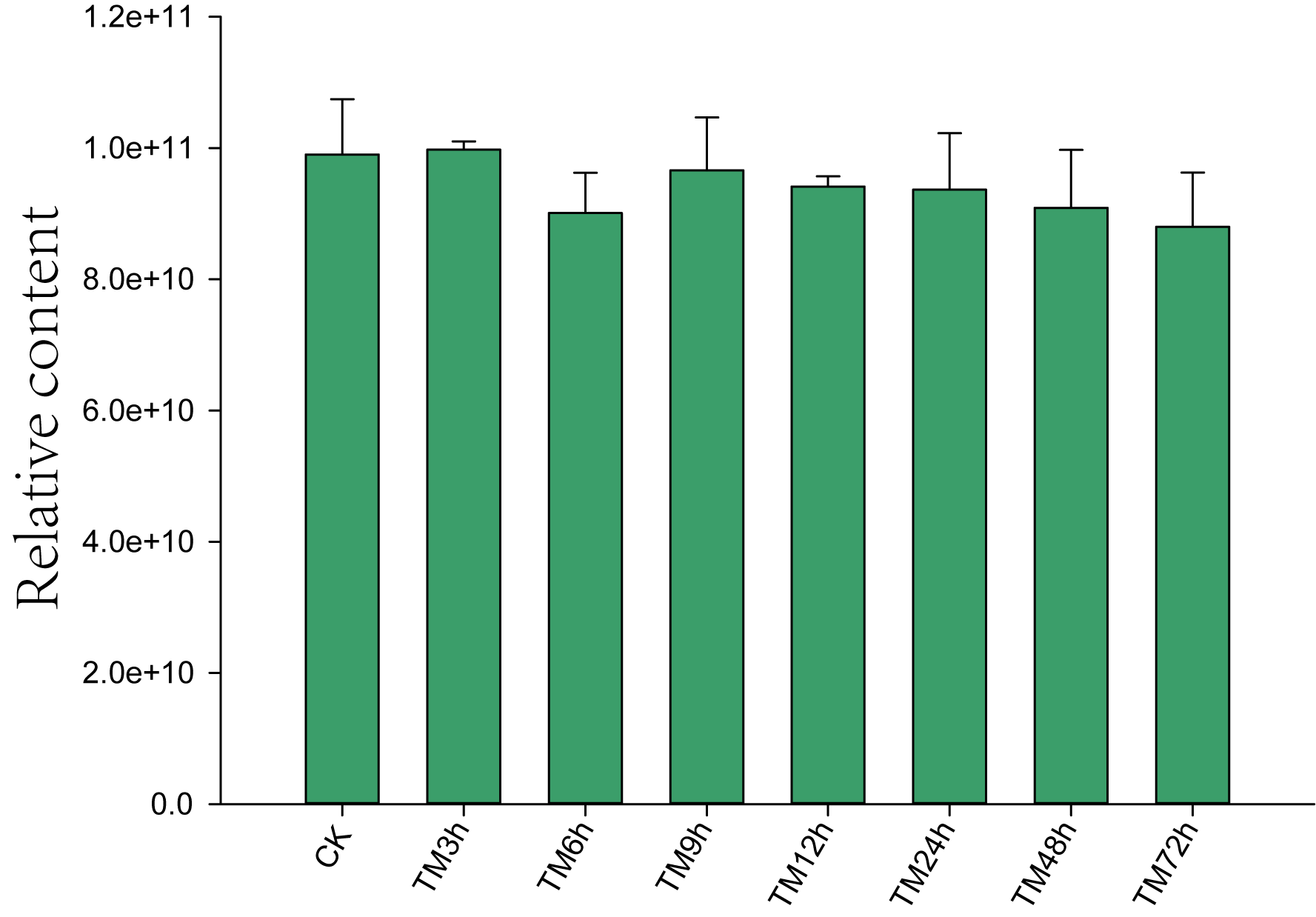

Supplement: Supplementary figure S2 — Relative contents of metabolites in samples at different time points. [file Image2.tif]

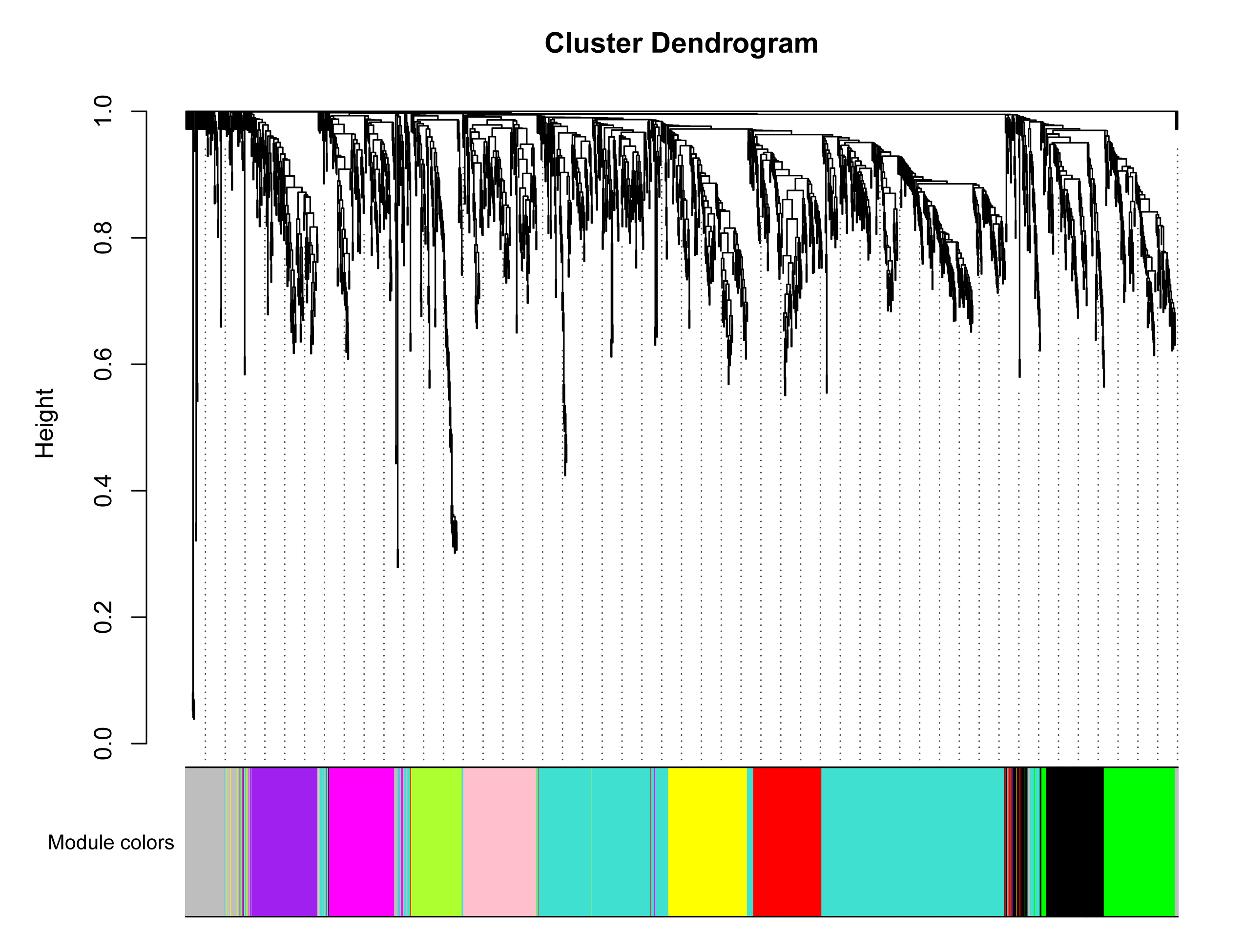

Supplement: Supplementary figure S3 — Gene clustering tree obtained from WGCNA analysis [file Image3.tif]
